# The Data Artifacts Glossary: a community-based repository for bias on health datasets

**DOI:** 10.1186/s12929-024-01106-6

**Published:** 2025-02-04

**Authors:** Rodrigo R. Gameiro, Naira Link Woite, Christopher M. Sauer, Sicheng Hao, Chrystinne Oliveira Fernandes, Anna E. Premo, Alice Rangel Teixeira, Isabelle Resli, An-Kwok Ian Wong, Leo Anthony Celi

**Affiliations:** 1 Laboratory for Computational Physiology, Massachusetts Institute of Technology, Cambridge, MA USA; 2 Department of Biostatistics, Harvard T.H. Chan School of Public Health, Boston, MA USA; 3 Laboratory for Clinical Research and Real-World Evidence, Department of Artificial Intelligence In Medicine, University Hospital Essen, Essen, Germany; 4 Department of Hematology and Stem Cell Transplantation, University Hospital Essen, Essen, Germany; 5 Division of Pulmonary, Allergy, and Critical Care Medicine, Duke University, Durham, NC USA; 6 Learning Research and Development Center, University of Pittsburgh, Pittsburgh, PA USA; 7 Department of Urban Studies and Planning, Massachusetts Institute of Technology, Cambridge, MA USA; 8 Department of Philosophy, Universitat Autónoma de Barcelona, Barcelona, Spain; 9 School of Electrical Engineering and Computer Science, Oregon State University, Corvallis, OR USA; 10 Department of Medicine, Beth Israel Deaconess Medical Center, Boston, MA USA

**Keywords:** Bias, Health equity, Dataset, Data Artifacts Glossary, Artificial intelligence, Machine learning

## Abstract

**Background:**

The deployment of Artificial Intelligence (AI) in healthcare has the potential to transform patient care through improved diagnostics, personalized treatment plans, and more efficient resource management. However, the effectiveness and fairness of AI are critically dependent on the data it learns from. Biased datasets can lead to AI outputs that perpetuate disparities, particularly affecting social minorities and marginalized groups.

**Objective:**

This paper introduces the “Data Artifacts Glossary”, a dynamic, open-source framework designed to systematically document and update potential biases in healthcare datasets. The aim is to provide a comprehensive tool that enhances the transparency and accuracy of AI applications in healthcare and contributes to understanding and addressing health inequities.

**Methods:**

Utilizing a methodology inspired by the Delphi method, a diverse team of experts conducted iterative rounds of discussions and literature reviews. The team synthesized insights to develop a comprehensive list of bias categories and designed the glossary’s structure. The Data Artifacts Glossary was piloted using the MIMIC-IV dataset to validate its utility and structure.

**Results:**

The Data Artifacts Glossary adopts a collaborative approach modeled on successful open-source projects like Linux and Python. Hosted on GitHub, it utilizes robust version control and collaborative features, allowing stakeholders from diverse backgrounds to contribute. Through a rigorous peer review process managed by community members, the glossary ensures the continual refinement and accuracy of its contents. The implementation of the Data Artifacts Glossary with the MIMIC-IV dataset illustrates its utility. It categorizes biases, and facilitates their identification and understanding.

**Conclusion:**

The Data Artifacts Glossary serves as a vital resource for enhancing the integrity of AI applications in healthcare by providing a mechanism to recognize and mitigate dataset biases before they impact AI outputs. It not only aids in avoiding bias in model development but also contributes to understanding and addressing the root causes of health disparities.

**Supplementary Information:**

The online version contains supplementary material available at 10.1186/s12929-024-01106-6.

## Introduction

Artificial Intelligence (AI) has the potential to revolutionize healthcare by offering sophisticated algorithms capable of diagnosing diseases, crafting personalized treatment plans, aiding clinicians in decision-making processes, and alleviating the administrative burden on healthcare practitioners [1, 2]. This technological advancement has been proposed to not only enhance the efficiency of healthcare delivery but also to shift the focus back to patient-centered care [3]. However, achieving this promise is not without its challenges. Deploying AI in healthcare presents complexities unparalleled in other sectors, primarily due to the intricate nature of medical practice, the historical biases ingrained within it and intrinsic epidemiological challenges working with electronic health record data [4].

Bias in research is defined as a systematic error or tendency that prevents impartial consideration by favoring one answer over others [5]. The issue of clinical bias is a well-known topic in the medical field, underscored by extensive research that explores its manifestations within a societal framework marked by inequity, prejudice, and discrimination [6]. These biases have tangible and detrimental effects on patient care, leading to disparities in diagnosis, treatment, and outcomes across diverse populations [7]. For instance, when compared to white Americans, pain management in black Americans is systematically worse, due to false beliefs about biological differences between these two groups [8]. Moreover, racial and ethnic minority patients are less likely to be screened for diabetic retinopathy, even though they are more likely to have poorer glycemic control [9].

Training AI on clinical data derived from a world rife with such biases risks not merely replicating, but also amplifying and perpetuating them [10, 11]. Notwithstanding, it could even reconfigure new ones that would remain elusive due to the inherently opaque nature of some AI algorithms [12]. Already, evidence of bias in AI spans across a variety of applications, from sex-based disparities in algorithms predicting cardiovascular risks [13], to ethnic disparities in the detection of skin-related diseases such as melanoma [14]. Notably, the issue of algorithmic bias extends beyond historically marginalized groups, potentially affecting anyone whose profile deviates from the predominant characteristics of the training datasets, whether in terms of skin color, gender, age, disease characteristics or even the hospital’s zip code [12].

Nevertheless, instead of viewing these biases purely as flaws, they can be seen as “Data Artifacts” —records of societal values, healthcare practices, and historical inequities. By examining biased clinical data through this lens, researchers can uncover underlying patterns of exclusion and injustice that persist in healthcare. As proposed by Ferryman et al. (2023), this artifact-based approach can help AI developers not only detect and avoid bias, but also understand the root causes of health inequities. Such an understanding is crucial for medical research overall, and especially for developing AI systems that do not merely replicate existing injustices but actively contribute to more equitable healthcare practices [15]. By treating biased data as informative artifacts, we can examine healthcare data more holistically, uncovering population inequities and suggesting novel uses of AI to detect health equity–relevant data patterns.

Furthermore, there has been growing awareness on the need for transparency and accountability for AI medical applications. With it, the investigation of bias in AI algorithms and medical devices is a rapidly advancing field. Recently, the European Parliament, through the EU-AI Act [16], the White House, via the Executive Order on the Safe, Secure, and Trustworthy Development and Use of Artificial Intelligence [17], and the US Department of Health and Human Services, through the final rule on section 1557 of the Affordable Care Act, have initiated measures to mitigate bias in AI algorithms. However, much of the effort in this domain has been directed towards post hoc analysis—examining models for bias after their development and deployment [18]. We see this approach as costly, inefficient, and unable to promote systemic change.

Some researchers have put forward commendable efforts aimed at enhancing the understanding of datasets’ collection processes, origins, development intents, recommended uses, and ethical considerations. These initiatives seek to establish standardized means for researchers and developers to quickly access critical information about datasets intended for training medical devices, algorithms, or conducting epidemiological research. Notable among these efforts are Data Cards [19], Data Statements [20], Datasheet for Datasets [21], Model Cards [22], AI-Usage Cards [23], and the Dataset Nutritional Label [18]. Each of these proposals contributes with valuable frameworks for documenting various aspects of datasets and models, facilitating a more responsible and informed use of data in AI development.

However, most existing initiatives primarily address general dataset characteristics and usage guidelines without delving deeper into the specific biases, or “artifacts” that datasets may contain. These existing frameworks, while foundational, are not equipped to dynamically track or update bias-related issues as they evolve or as new evidence comes to light. Moreover, they fall short of providing the nuanced understanding required to preemptively recognize and investigate biases specific to each dataset. This oversight underscores the critical need for a community-based repository that systematically indexes, catalogs, and describes biases as informative data artifacts.

We propose the development of the *Data Artifacts Glossary*—a dynamic, open-source framework that serves as a collaborative platform for examining healthcare data bias as artifacts. By expanding the technical approach to data bias in AI development to include sociotechnical perspectives, the Glossary considers historical and current social contexts as important factors in addressing bias. This expanded approach not only aids in avoiding bias in model development but also serves the public health goal of understanding population inequities and suggests novel uses of AI to detect health equity–relevant data patterns.

## Methods

The development of the *Data Artifacts Glossary* was guided by a methodology inspired by the Delphi method [24], a structured communication technique well-suited for achieving consensus among a diverse group of experts. Our team consisted of clinicians, computer scientists, data scientists, researchers, project managers, specialists in education, and legal experts. The process began with an initial round of discussions where each team member independently provided their insights on the potential sources of bias in healthcare datasets. These insights were compiled into a preliminary list of bias types and framework concepts. To ensure a comprehensive approach, we conducted an extensive literature review to identify existing frameworks and data documentation methods, including examining Data Cards, Data Statements, Datasheet for Datasets, Model Cards, AI-Usage Cards, and the Dataset Nutritional Label.

Following this, several rounds of structured discussions were conducted, each designed to refine and expand upon the preliminary concepts. These rounds involved feedback and structured iterations, facilitating a thorough examination and synthesis of diverse perspectives. Through this iterative process, the team reached a consensus on the most pertinent categories of bias to include in the glossary. Each category was chosen based on its relevance to clinical data and its potential impact on AI applications. Additionally, considerable discussion was dedicated to designing the structure of the glossary itself. Finally, the methodology included pilot testing the glossary with the MIMIC-IV dataset to validate its structure and utility. The pilot involved a detailed review of the dataset’s published literature to identify and document specific biases, followed by the incorporation of this information into the glossary framework.

## Results

The *Data Artifacts Glossary* is envisioned as a collaborative platform designed to systematically document and update biases associated with both public and non-public healthcare datasets. Unlike existing frameworks that provide static snapshots of data characteristics, this *Glossary* aims to establish a dynamic, community-driven repository where biases are continually identified, reported, and potential mitigation strategies revised. By viewing biased clinical data as informative artifacts, the *Data Artifacts Glossary* facilitates a deeper examination of societal values, healthcare practices, and historical inequities reflected in the data.

This living document will serve as a comprehensive reference point for researchers, clinicians, and AI developers, allowing them to understand not only the general attributes of a dataset but also the specific biases it may harbor. By integrating contributions from a diverse community of stakeholders, the *Data Artifacts Glossary* will evolve with the expanding landscape of medical data and emerging insights into biases, ensuring that the information remains current and relevant.

The *Data Artifacts Glossary* will adopt a collaborative model inspired by renowned open-source software practices, similar to those used by projects like Linux and Python. This approach will incorporate several key practices that have contributed to the success and widespread adoption of these software projects: Version Control, Public Reviews, and Documentation. First, the glossary will use a robust version control system at first facilitated by GitHub, a robust platform renowned for its strong collaborative features. This will allow multiple community members—including researchers, clinicians, AI developers, and other stakeholders—to simultaneously work on the glossary, efficiently tracking changes and managing versions. This transparent process ensures that every modification to the glossary’s codebase is well-documented and accessible. Second, modifications and enhancements to the glossary will be handled through “pull requests”. These requests, which community members can submit, are essentially proposals for revisions or additions to the glossary. Each pull request is made available publicly for review, fostering a rigorous peer review process. This process is managed by project maintainers who are selected based on their expertise and commitment to promoting unbiased AI in healthcare. The peer review ensures that all contributions adhere to high standards of quality and functionality.

**Fig. 1:**
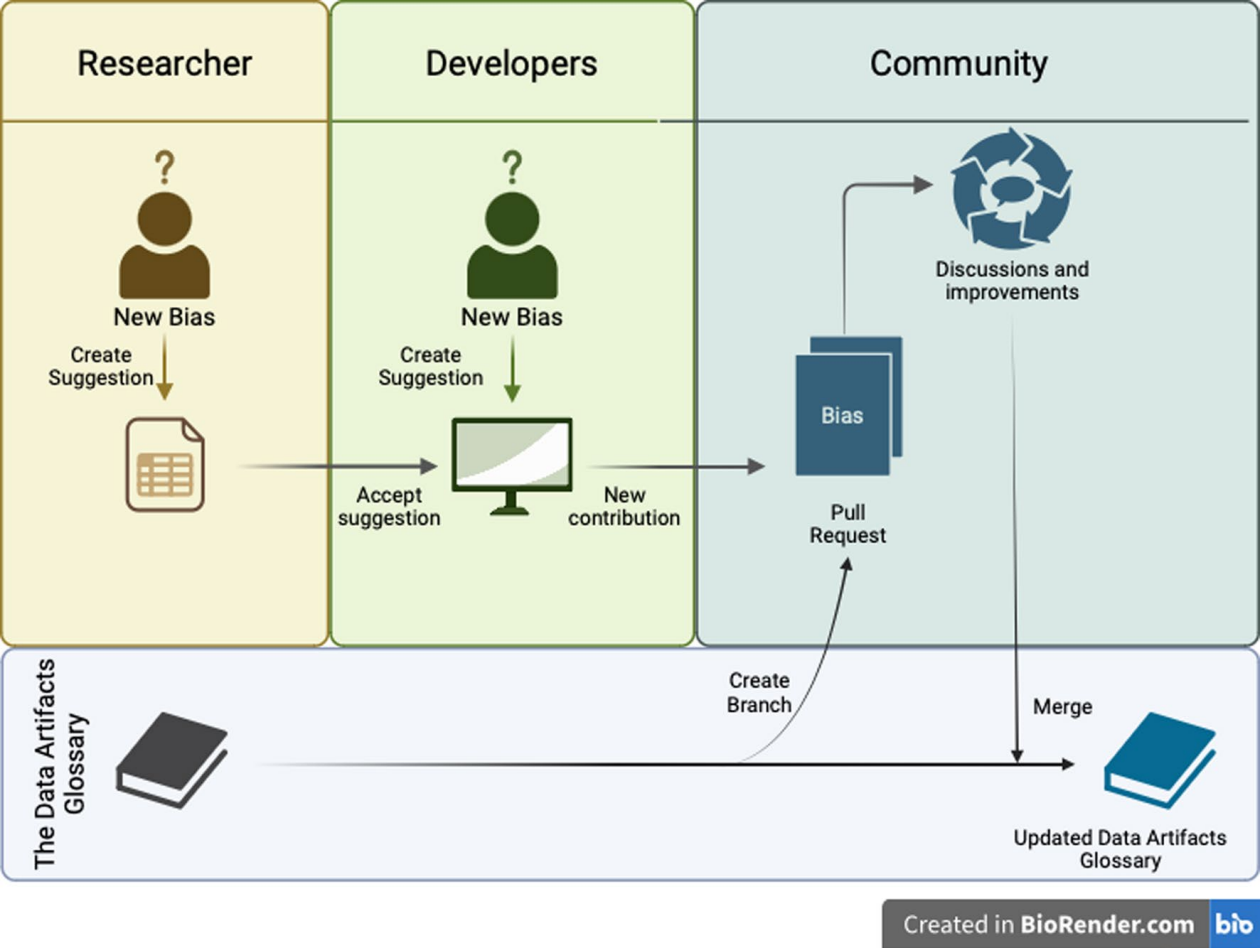
Workflow diagram illustrating the collaborative process of the *Data Artifacts Glossary*. Researchers and developers can suggest new biases which are then reviewed and potentially accepted by the community. Once approved, these suggestions are merged back into the *Data Artifacts Glossary,* ensuring it remains an up-to-date and evolving resource

Lastly, comprehensive documentation will be a cornerstone of the *Data Artifacts Glossary* project. Effective documentation is vital in open-source projects as it helps new users understand how to utilize the tool and aids new contributors in grasping the codebase and the project’s architecture. To allow contributions from researchers or community members who may not be familiar with coding, we introduced a more intuitive feature for collaboration. These users can access the *Data Artifacts Glossary* on GitHub via a provided link and submit suggestions through a user-friendly form (detailed in the “*Data Artifacts Glossary* Contribution Guide” tab). Developers can then review and incorporate these suggestions into the *Data Artifacts Glossary*, fostering broader participation and diverse input. Figure 1 provides a clear visual representation of the collaborative workflow and its components.

This methodology aims to foster an ongoing, dynamic update process and ensure that the glossary maintains a high level of academic rigor. The open-source model is designed to promote inclusivity and collective responsibility, essential for addressing the multifaceted nature of biases in healthcare datasets. By leveraging the collective intelligence of an interdisciplinary community, the *Glossary* facilitates the examination of biases as artifacts within their broader social and historical contexts, promoting a deeper understanding of the root causes of health inequities. This approach mirrors the principles of openness, peer review, and community engagement that are hallmarks of both academic rigor and the key practices of open-source projects.

The platform also features detailed documentation on each dataset, including its origin, collection process, and any amendments made to its associated biases, thereby providing transparency and traceability. In essence, the *Data Artifacts Glossary* will act as both a repository and a forum, fostering a collaborative environment for sharing knowledge and best practices in addressing dataset biases.

The ultimate goal of the *Data Artifacts Glossary* is to enhance the integrity and efficacy of AI applications in healthcare by providing a resource that helps mitigate the risk of bias from the beginning. By equipping stakeholders with detailed, up-to-date information on dataset biases, the Glossary aims to aid in the development of more accurate and fair AI algorithms. More importantly, by viewing biases as informative artifacts, the *Glossary* helps uncover and address the root causes of healthcare inequities, offering a more holistic approach to ethical AI use. It also aims to support the broader objective of ethical AI use, aligning with international efforts to ensure that AI systems are safe, secure, and trustworthy.

### Suggested first version for MIMIC-IV

**Fig. 2:**
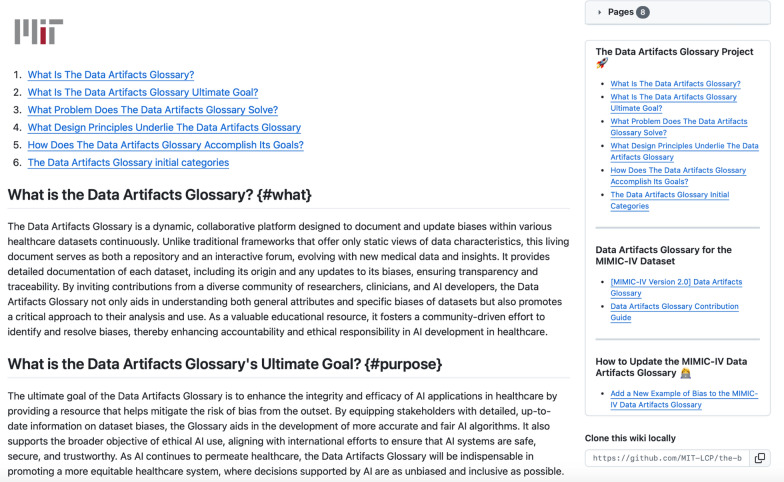
Screenshot of the main page of the *Data Artifacts Glossary*. This page provides an overview of the project's goals, design principles, and initial categories, as well as links to detailed descriptions and guides for contributing to the *Data Artifacts Glossary* for the MIMIC-IV dataset

**Fig. 3:**
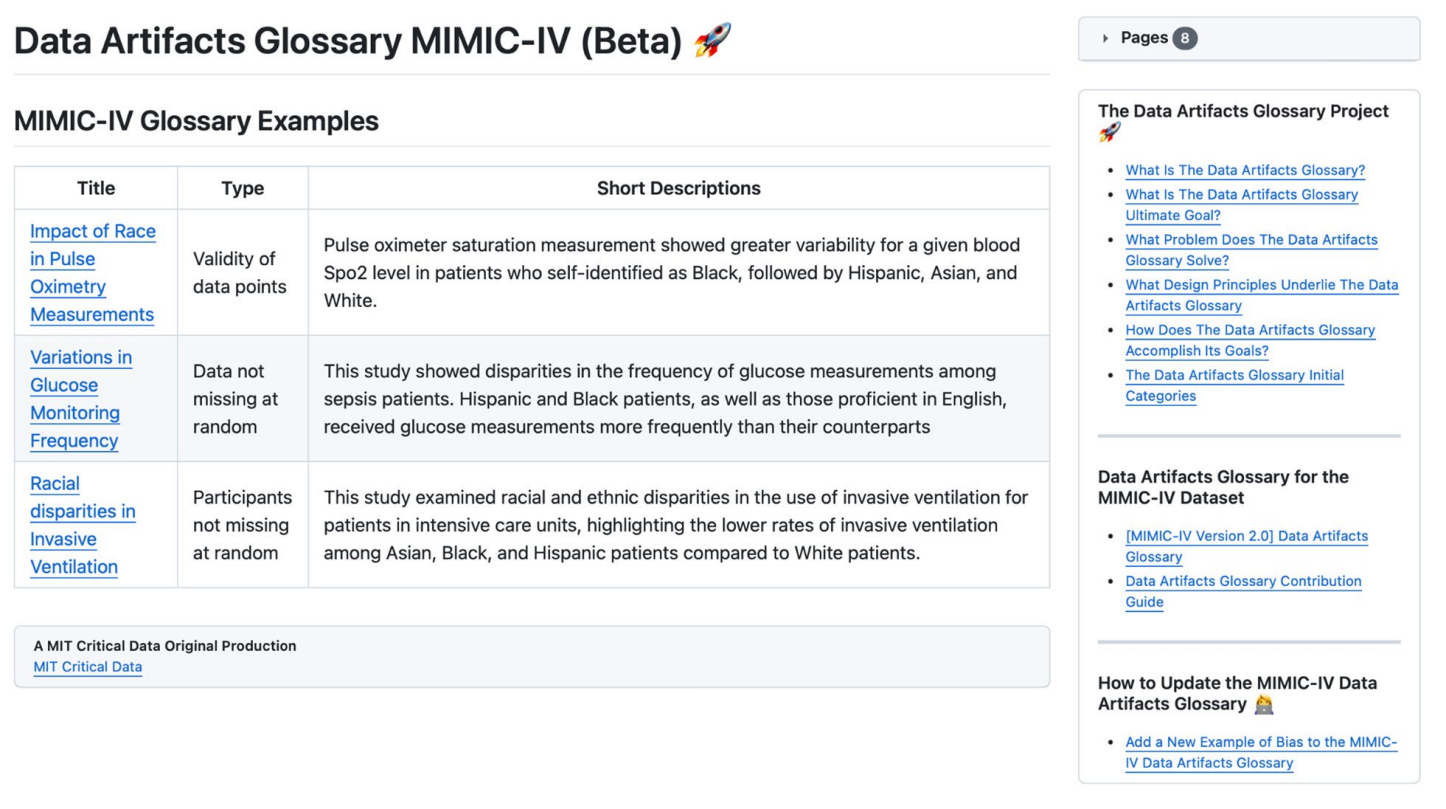
Screenshot of the Data Artifacts Glossary for the MIMIC-IV (Beta)

**Fig. 4:**
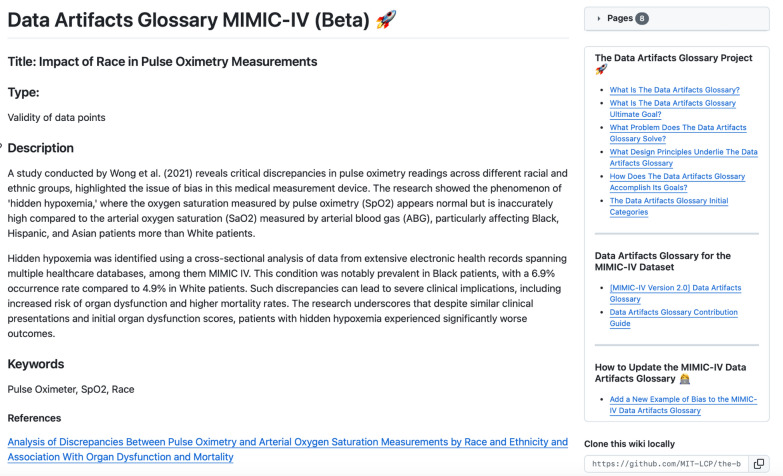
Screenshot of detailed bias entry for the Data Artifacts Glossary for the MIMIC-IV (Beta)

To demonstrate the practical application and utility of the *Data Artifacts Glossary*, we initiated a beta version of this platform (Figs. 2, 3, and 4) using the Medical Information Mart for Intensive Care (MIMIC-IV) dataset [25]. This dataset, consisting of de-identified health data associated with over seventy thousand patients admitted to critical care units of the Beth Israel Deaconess Medical Center in Boston, Massachusetts, is widely used for research in various domains of healthcare.

Here, we aim to suggest one potential structure as a starting point to populate the *Data Artifacts Glossary,* consisting of four initial categories, namely: Participants not missing at random, Validity of data points, Data not missing at random, and Miscellaneous. Of note, we do not aim to provide the final structure of the *Data Artifacts Glossary*, nor do we claim that the following categories are exhaustive.

### Participants not missing at random

This category captures bias stemming from absence or underrepresentation of specific patient groups within the dataset, encompassing not only demographic factors but also clinical conditions, socioeconomic statuses, and accessibility variables which may skew research outcomes and subsequent clinical applications. The *Data Artifacts Glossary* under this category aims to illuminate the hidden disparities by documenting the absence of certain groups due to various selection biases or data collection constraints. This awareness is critical as it allows researchers and clinicians to critically evaluate the dataset and its applicability to the target patient population, ensuring that medical interventions developed from AI models do not inadvertently perpetuate health inequities. For example, a study using MIMIC-IV data found that Asian, Black, and Hispanic patients received invasive ventilation at significantly lower rates than White patients, despite presenting with similar clinical severity [26], indicating a potential systemic bias in treatment practices across racial lines. This discrepancy may be due to implicit biases in clinical decision-making, or differences in how symptoms are assessed and acted upon for patients of different ethnicities.

### Validity of data points

The second category examines the integrity of data collected, focusing on potential biases introduced through the use of various medical devices and data recording methodologies. This category is pivotal as it questions the foundational accuracy of the dataset itself —whether the data points reflect true patient states or are distorted by technological and procedural variances. By cataloging these potential sources of error, the *Data Artifacts Glossary* promotes a more nuanced understanding of the data, which is essential for developing reliable AI models. For instance, a study using MIMIC-IV found that hidden hypoxemia was more frequently under-detected in Black and Hispanic patients [27], underscoring the crucial bias in the accuracy of pulse oximetry measurements across different racial groups. This issue may have arisen because pulse oximeters were predominantly developed and calibrated using lighter-skinned populations, leading to decreased accuracy in individuals with darker skin pigmentation and underestimation of oxygen deprivation in these patients.

### Data not missing at random

This category investigates the uneven data collection practices that may occur across various patient groups due to factors such as race, socioeconomic status, geographical location, and other demographic or contextual influences. It underscores the necessity to meticulously examine and question the consistency and fairness of data collection protocols and their execution among diverse patient populations. This detailed scrutiny is crucial for identifying and understanding the systemic errors and biases that could detrimentally impact clinical research and the training of AI algorithms. Under this category, the MIMIC-IV *Data Artifacts Glossary* lists the discrepancy between observed glucose measurement frequencies among different demographic groups, where significant increases in measurement frequency were found for individuals identified as male, Hispanic, Black, or English proficient [28]. One hypothesis is the presence of language barriers, making it more challenging for providers to communicate with non-English proficient patients to explain procedures, potentially leading to fewer glucose measurements being performed for these patients.

### Miscellaneous biases

The fourth category encompasses a broad range of biases that do not neatly fit into the other categories but are nonetheless crucial for understanding and using the dataset responsibly. These might include biases related to the geographic location of data collection, time-period specific healthcare practices, or administrative biases in how data are recorded and processed. This section will be populated with examples that highlight impactful biases affecting data interpretation and application in AI systems.

The current glossary is not exhaustive and not static. New causes of bias having profound effects on downstream prediction, classification and optimization tasks will continuously be found for various datasets. From differential performance of medical devices used to measure physiologic signals across patient populations, to variation in the frequency of testing across patient populations that is not explained by clinical factors, to disparities in the performance of routine care that is typically assumed to be administered uniformly across patient populations. These discoveries are made possible by a collaborative community of users who are curating and analyzing its data and sharing those discoveries with each other. In the case of MIMIC, we expect the 70 k + users to contribute insights on sources of bias captured in the first *Data Artifacts Glossary*. We hope this serves as a lighthouse to establish more bias glossaries for other public and non-public datasets.

## Discussion

The *Data Artifacts Glossary* represents a transformative approach to collaboratively identify and understand biases within healthcare datasets, not by merely viewing biases as flaws to be corrected, but by recognizing them as informative artifacts that reflect societal values, healthcare practices, and historical inequities. By fostering an environment where biases are continuously identified, documented, and addressed through community-driven efforts, this living document not only enhances the integrity of AI applications in healthcare but also promotes a more equitable healthcare system. The adoption of open-source principles and robust peer-review mechanisms ensures that the Glossary remains an up-to-date, transparent, and reliable resource, pivotal for developing AI tools that are both effective and fair. As we stand on the brink of a new era in healthcare, marked by technological advancements, the *Data Artifacts Glossary* serves as a crucial tool to ensure that these technologies benefit all segments of society equally, preventing the perpetuation of historical inequities.

The practical implementation of the *Data Artifacts Glossary* using the MIMIC-IV dataset demonstrates its significant potential in improving the quality of AI in healthcare. By meticulously categorizing and documenting specific biases within this widely-used dataset, the Glossary enables researchers and clinicians to better understand and address the inherent biases present in the data. This understanding is critical, as it allows for the identification and potential rectification of biases before they influence AI models, which could otherwise perpetuate or even amplify existing health disparities. Using the MIMIC-IV *Data Artifacts Glossary* as an example, if O2 saturation is an important feature for developing a model, a researcher might choose to use arterial blood gas measurements instead of pulse oximetry. The *Data Artifacts Glossary* serves as both a reference and a guide, educating users about the various forms of bias and their implications, thus fostering a more informed and proactive approach to data management in healthcare AI.

Moreover, the collaborative nature of the *Data Artifacts Glossary* leverages the collective intelligence of a diverse group of stakeholders, including researchers, clinicians, and AI developers. This inclusive approach ensures that the Glossary remains comprehensive and reflects a wide range of perspectives, making it a robust tool for enhancing the fairness and accuracy of AI applications in healthcare. The community-driven contributions and rigorous peer review process ensure high-quality, reliable updates, keeping the Glossary relevant in a rapidly evolving field.

### Limitations

Despite its strengths, the *Data Artifacts Glossary* is not without limitations. One of the primary challenges lies in ensuring widespread and consistent participation from the community. The quality and usefulness of the glossary depend heavily on the contributions of its users, which can be variable and influenced by individual biases and expertise levels. Additionally, while the glossary provides a framework for documenting biases and lists potential mitigation strategies, it cannot offer direct solutions to mitigate all sources of bias, requiring users to choose and apply their own methods and work-arounds. Addressing these limitations requires ongoing efforts to engage the community, streamline contributions, address sources of bias upon data generation, and perhaps develop additional tools and guidelines for bias mitigation.

## Conclusion

The practical implementation of the *Data Artifacts Glossary*, demonstrated through the MIMIC-IV dataset, highlights its potential to significantly impact healthcare outcomes by providing a deeper, more nuanced understanding of dataset biases. This initiative is not merely a response to the growing complexity of medical datasets but a proactive measure to safeguard against the inadvertent introduction of biases by AI systems. By equipping researchers, clinicians, and policymakers with the knowledge to scrutinize and refine the datasets that train AI, the Glossary aids in the creation of more accurate and impartial medical AI applications. Furthermore, it serves as a novel tool for using AI to detect health equity–relevant data patterns, thereby expanding the potential of AI in promoting health equity. Moving forward, as the *Data Artifacts Glossary* continues to evolve, it will remain a vital resource for enhancing the fairness and accuracy of AI in healthcare, ensuring that it adapts to new challenges and insights in a rapidly advancing field.

## Supplementary Information

Additional file 1.

## Data Availability

Github code and wiki: https://github.com/MIT-LCP/the-bias-glossary/wiki.
