# Supplementary material for: The Data Artifacts Glossary: a community-based repository for bias on health datasets
Source: J Biomed Sci. 2025 Feb 4;32:14. doi: 10.1186/s12929-024-01106-6 (PMC11792693; doi:10.1186/s12929-024-01106-6)
Supplement: Supplementary file 1 — Additional file 1. [file 12929_2024_1106_MOESM1_ESM.docx]

**Appendix:**

Github code and wiki for the Data Artifacts Glossary: [**https://github.com/MIT-LCP/the-bias-glossary/wiki**](https://github.com/MIT-LCP/the-bias-glossary/wiki)
